# High prevalence of ceftriaxone-resistant and XDR *Neisseria gonorrhoeae* in several cities of Cambodia, 2022–23: WHO Enhanced Gonococcal Antimicrobial Surveillance Programme (EGASP)

**DOI:** 10.1093/jacamr/dlae053

**Published:** 2024-04-04

**Authors:** V Ouk, L Say Heng, M Virak, S Deng, M M Lahra, R Frankson, K Kreisel, R McDonald, M Escher, M Unemo, T Wi, I Maatouk, EGASP Cambodia Working Group, Phnom Penh, Vivian Fensham, Ellen Kersh, Philippe Cavailler, Yamuna Mundade, Sebastiaan J van Hal, Ratan L Kundu, Tiffany R Hogan, David M Whiley, Kiyohiko Izumi, Takeshi Nishijima

**Affiliations:** National Center for HIV/AIDS, Dermatology and Sexually Transmitted Diseases, Phnom Penh, Cambodia; National Center for HIV/AIDS, Dermatology and Sexually Transmitted Diseases, Phnom Penh, Cambodia; Laboratory of the National Institute of Public Health, Phnom Penh, Cambodia; WHO, Office of Cambodia, Phnom Penh, Cambodia; WHO Collaborating Centre for Sexually Transmitted Infections and Antimicrobial Resistance, New South Wales Health Pathology, Microbiology, The Prince of Wales Hospital, Randwick, NSW, Australia; Division of STD Prevention, CDC, Atlanta, GA, USA; Division of STD Prevention, CDC, Atlanta, GA, USA; Division of STD Prevention, CDC, Atlanta, GA, USA; AMR Division, WHO, Geneva, Switzerland; Department of Laboratory Medicine, Faculty of Medicine and Health, WHO Collaborating Centre for Gonorrhoea and other STIs, Örebro University, Örebro, Sweden; Institute for Global Health, University College London, London, UK; Department of the Global HIV, Hepatitis and STI Programmes, WHO, Geneva, Switzerland; Department of the Global HIV, Hepatitis and STI Programmes, WHO, Geneva, Switzerland

## Abstract

**Objectives:**

Antimicrobial resistance (AMR) in *Neisseria gonorrhoeae* is a global public health concern. Ceftriaxone is the last effective and recommended option for empirical gonorrhoea therapy worldwide, but several ceftriaxone-resistant cases linked to Asia have been reported internationally. During January 2022–June 2023, the WHO Enhanced Gonococcal Antimicrobial Surveillance Programme (EGASP) investigated *N. gonorrhoeae* AMR and epidemiological factors in patients from 10 clinical sentinel sites in Cambodia.

**Methods:**

Urethral swabs from males with urethral discharge were cultured. ETEST determined the MIC of five antimicrobials, and EGASP MIC alert values and EUCAST breakpoints were used. EGASP demographic, behavioural and clinical variables were collected using a standardized questionnaire.

**Results:**

From 437 male patients, 306 had positive *N. gonorrhoeae* cultures, AMR testing and complete epidemiological data. Resistance to ceftriaxone, cefixime, azithromycin and ciprofloxacin was 15.4%, 43.1%, 14.4% and 97.1%, respectively. Nineteen (6.2%) isolates were resistant to all four antimicrobials and, accordingly, categorized as XDR *N. gonorrhoeae*. These XDR isolates were collected from 7 of the 10 sentinel sites. No EGASP MIC alert values for gentamicin were reported. The nationally recommended cefixime 400 mg plus azithromycin 1 g (65.4%) or ceftriaxone 1 g plus azithromycin 1 g (34.6%) was used for treatment.

**Conclusions:**

A high prevalence of ceftriaxone-resistant, MDR and XDR *N. gonorrhoeae* in several cities of Cambodia were found during 2022–23 in WHO EGASP. This necessitates expanded *N. gonorrhoeae* AMR surveillance, revision of the nationally recommended gonorrhoea treatment, mandatory test of cure, enhanced sexual contact notification, and ultimately novel antimicrobials for the treatment of gonorrhoea.

## Introduction

The WHO estimated that in 2020 approximately 82 million new cases of gonorrhoea among adults occurred worldwide.^1^ Urogenital gonorrhoea typically presents as urethritis or cervicitis that, if untreated, can lead to serious complications like pelvic inflammatory disease, ectopic pregnancy and infertility. Antimicrobial resistance (AMR) in *Neisseria gonorrhoeae* to all antimicrobials introduced for empirical treatment of gonorrhoea has been developed or acquired.^2,3^ Resistance has now emerged to the last-line option for empirical treatment of gonorrhoea, i.e. ceftriaxone, which frequently is administered together with azithromycin. This is a global public health concern as declared by the WHO, especially since 2012 when the first occasional ceftriaxone-resistant *N. gonorrhoeae* strains had started to spread and consequently the WHO global action plan to control the spread and impact of AMR in *N. gonorrhoeae* was published.^4^ Sporadic treatment failures with ceftriaxone have been confirmed in many countries.^3^ It is also a major concern that since 2015 an international spread of the ceftriaxone-resistant FC428 clone has been documented^3,5–7^ and this strain appears to now be in sustained transmission, i.e. acting as a reservoir, in several Asian countries such as Japan and China.^6^ Furthermore, the first two strains with resistance to ceftriaxone combined with high-level azithromycin resistance (MIC ≥ 256 mg/L) were identified in 2018 (the same strain in the UK and Australia)^8^ and 2022 (in Austria).^9^ The vast majority of ceftriaxone-resistant isolates have been linked to Asia, either by being cultured in Asia or cultured in patients with infection likely acquired in Asia.^3,5–9^ Some of these ceftriaxone-resistant isolates have been linked to Cambodia. For example, the second global strain with ceftriaxone resistance combined with high-level resistance to azithromycin was detected in Austria, but the patient acquired infection following a sexual encounter with a female sex worker in Cambodia.^9^ Additionally, sporadic isolates of ceftriaxone-resistant isolates cultured in France^10,11^ and Australia^7^ were also linked to recent travel to Cambodia.

Cambodia has recently slowed down its national HIV epidemic by reaching promising levels of the three UNAIDS targets, achieving 86%-99%-98% by the end of 2022.^12^ Despite this dramatic decrease in HIV prevalence in the general population, the HIV epidemic remains highly concentrated among key populations.^12^ Moreover, very limited data regarding the prevalence and epidemiology of sexually transmitted infections (STIs) in Cambodia have been published. An integrated HIV biobehavioural survey among female sex workers conducted in 2022 reported a prevalence of *N. gonorrhoeae* of 18.0%, *Chlamydia trachomatis* 22.6%, syphilis 18.9% and HIV 4.9%.^13^ A similar study conducted in 2019 among MSM reported a prevalence of *N. gonorrhoeae* of 4%, *C. trachomatis* 7.1% and syphilis 6%.^14^ No comprehensive *N. gonorrhoeae* AMR surveillance has been previously performed in Cambodia.

WHO’s global Gonococcal Antimicrobial Surveillance Programme (GASP) was established in 1990 as a global surveillance laboratory network.^15^ WHO GASP has monitored global *N. gonorrhoeae* AMR, detected emerging AMR, and informed revisions of national and international treatment guidelines.^3,16,17^ However, WHO GASP includes limitations such as lack of standardization, quality assurance and epidemiological data of patients, and to address these limitations and enhance the global *N. gonorrhoeae* AMR surveillance, WHO Enhanced GASP (EGASP) was launched.^18,19^

WHO EGASP operates under the umbrella of the WHO Global Antimicrobial Resistance and Use Surveillance System (GLASS), and it is a collaboration involving the WHO, the Division of STD Prevention (DSTDP) at the US CDC, WHO Collaborating Centres and the EGASP countries. The primary objective is to monitor trends in *N. gonorrhoeae* AMR in men with urethral discharge, using standardized sampling and quality-assured laboratory protocols in selected EGASP sentinel countries worldwide. The ultimate goal is to improve the early detection and reporting of *N. gonorrhoeae* strains with resistance to internationally recommended treatment for gonorrhoea. The general WHO EGASP protocol was published online in 2021.^19^

The objective of the present paper is to describe the first results of the WHO EGASP surveillance in Cambodia from January 2022 (date of EGASP implementation in Cambodia) to June 2023, to substantially expand on a preliminary WHO EGASP report indicating the presence of ceftriaxone-resistant *N. gonorrhoeae* strains in Cambodia.^20^

## Materials and methods

### EGASP surveillance sites and participants

The EGASP activities in Cambodia are managed by the National Center for HIV/AIDS, Dermatology, and STD (NCHADS), with the National Institute of Public Health (NIPH) in Phnom Penh serving as the reference laboratory. From January 2022, 10 clinical sentinel sites enrolled EGASP participants: six sites are in the capital city Phnom Penh [Family Health Clinic Samdach Ov, Family Health Clinic Chactomok, Family Health Clinic Pochentong, Family Health Clinic Turl Kok, Chhouk Sar Clinic and National Clinic for Dermatology and STDs (NCDS)], two sites in the Prey Veng province (Family Health Clinic and Family Health Clinic Neak Leong) and one site each in the Kampong Speu province (Family Health Clinic) and Kampong Cham province (Family Health Clinic). These clinics typically specialize in sexual health and STIs and are primarily outpatient facilities.

Consecutive men attending the clinical sentinel sites with urethral discharge are enrolled, and each man is included only once per gonorrhoea episode.

### Demographic, behavioural and clinical data

A standardized abstraction form developed by the EGASP team in Cambodia is used to collect demographic, behavioural and clinical data. These data include age, type of patient (new patient versus established patient with repetitive episodes), symptoms of gonorrhoea, primary and any secondary gonorrhoea treatment, outcome of treatment (treatment completed or partial), outcome of any follow-up visit (symptoms or asymptomatic), result of any test of cure (TOC), antibiotic use in the previous 2 weeks, history of travel within the past 30 days, gender of sexual partners in the past 30 days (sex with men, women or both), number of sexual partners in the past 30  days, sexual behaviours in the past 30 days (vaginal, anal and oral sex) and coinfections with other STIs including HIV.

The collected data, along with the collected EGASP biological specimen(s), were then transferred to the NIPH reference laboratory where all data were linked to laboratory data, such as culture and antimicrobial susceptibility testing (AST) results.

### Clinical management

In the clinical sentinel sites, all individuals presenting with urethral discharge were provided with same-day syndromic management according to the Cambodian National STIs guidelines (2019).^21^ The recommended first-line empirical treatment is cefixime 400 mg plus azithromycin 1 g, single oral dose for both.^21^

Patients were also advised to attend a follow-up visit, especially if the symptoms persisted for 7 days after the treatment and/or if the *N. gonorrhoeae* isolates obtained from the patient’s samples exhibited EGASP MIC alert or high alert values for any of the tested antibiotics. Additionally, counselling about the importance of sexual contact notification and treatment for urethritis was provided.

### Specimen collection and laboratory procedures

Urethral swab specimens were sampled and stored in containers with ice packs (2°C–8°C) prior to transportation to NIPH, which was performed within 6 h of specimen collection. At NIPH, the urethral swabs were promptly inoculated on selective culture medium (modified Thayer–Martin), within 1–2 h the inoculated culture plates were incubated at 35°C–36.5°C in a 5% CO_2_-enriched atmosphere, and the culture plates were subsequently examined after 24 and 48 h of incubation. Presumptive identification of *N. gonorrhoeae* was based on the growth of typical colonies on the selective culture medium, a rapid positive oxidase test, and the observation of Gram-negative diplococci in microscopy. The isolates were preserved in duplicate at −70°C prior to AST.

MICs (mg/L) of ceftriaxone, cefixime, azithromycin, ciprofloxacin and gentamicin were determined using ETEST (bioMérieux, Marcy-l'Étoile, France), in accordance with the manufacturer’s instructions. The 2016 WHO *N. gonorrhoeae* reference strains WHO K or WHO L^22^ (alternatively for each test run) were used for quality control of the AST. Annual external quality assessments (EQAs) by CDC and the WHO Collaborating Centre for STI and AMR, New South Wales (NSW) Health Pathology, Australia were performed and both these EQAs were successful in 2022. The MIC values were interpreted using EGASP MIC alert and EGASP MIC high alert values^19^ and the EUCAST v13.1 clinical breakpoints (Table 1).^23^ EUCAST does not state any clinical resistance breakpoints for azithromycin and gentamicin. However, the epidemiological cut-off (ECOFF) for azithromycin (MIC > 1 mg/L)^23^ was used to indicate azithromycin resistance (referred to as resistant below). MDR and XDR *N. gonorrhoeae* were defined based on a modified version^24^ of the original definitions published by Tapsall *et al*.^25^

**Table 1.** MIC breakpoint values used in the present study

Antibiotic	Alert value, mg/L (WHO EGASP^19^)	High alert value, mg/L (WHO EGASP^19^)	Resistance breakpoints, mg/L (EUCAST^23^)
Ceftriaxone	≥0.125	≥0.5	>0.125
Cefixime	≥0.25	≥0.5	>0.125
Azithromycin	≥2.0	≥256^b^	>1.0
Gentamicin^a^	≥32.0	NA	NA
Ciprofloxacin^a^	≥1.0	NA	>0.06

NA, not applicable.

^a^Optional to test in WHO EGASP.

^b^Recognized as high-level resistance to azithromycin.

All isolates with an EGASP MIC alert/high alert value^19^ (including all isolates resistant according to the EUCAST breakpoints^23^) were confirmed by repeated MIC testing. Once an EGASP MIC alert value was confirmed, the EGASP national focal point, the EGASP coordinator and the respective sentinel clinic were promptly informed either via telephone or e-mail. In cases where isolates exhibited EGASP MIC high alert values, immediate retesting (urethral culture) was initiated, and this information was promptly (prior to retesting) conveyed to the WHO, CDC and the supporting WHO Collaborating Centre.

### Data management and validation

To ensure comprehensive data collection and validation, all patient and laboratory data were sent to the EGASP national coordinator within 2 months after the end of the month of isolate collection. Rigorous validation processes were implemented to ensure high data quality.

To facilitate secure and anonymous data entry, management and validation, an EGASP data entry form has been developed within the WHO EGASP dedicated platform.^19^ Given the potential sensitivity of the surveillance data, both paper-based and electronic data must be stored securely, with access restricted to authorized personnel only. However, data uploaded on the dedicated EGASP platform is completely anonymous.

### Statistical analyses

SPSS version 28.0 (IBM Corporation, NY, USA) was used for data analysis. Descriptive statistical analyses were conducted to compute medians, means and standard deviations (SDs) for numerical variables and frequencies (%) for categorical variables.

### Ethics

The protocol of WHO EGASP was reviewed by the Research Ethics Review Committee of the WHO (Protocol ID: EGASPprotocolGen), the National Health Technical Committee in Cambodia, CDC, and confirmed as a public health surveillance protocol (not a research protocol). Accordingly, WHO EGASP surveillance was exempt from full ethical review and considered as routine surveillance. Notably, no patient identifiable data are transmitted since all records contain a non-identifiable unique identification number (EGASP ID). Data collected and stored at the clinical sentinel sites and NIPH are secured and kept confidential according to local standard operating procedures.

## Results

### Demographic and behavioural characteristics

From January 2022 to 30 June 2023, there were 437 episodes of urethral discharge in men who presented to the 10 clinical sentinel sites, among whom 414 (94.7%) had complete epidemiological and laboratory data. A total of 308 *N. gonorrhoeae* isolates were cultured from these EGASP cases and AST results were available for 306 (99.4%) of these isolates.

The mean (median) age of these 306 EGASP participants with complete epidemiological data and *N. gonorrhoeae* isolates was 27.8 years, and the most common age group was 25–34 years (45.1%). Overall, 50% reported to be MSM, 1.6% reported antibiotic use within the past 2 weeks, 32.0% had a recent travel history (all only within Cambodia) and 5.3% had six or more sexual partners within the past 30 days. The demographic and behavioural characteristics of the EGASP participants with positive *N. gonorrhoeae* culture have been summarized in Table 2.

**Table 2.** Demographic and behavioural characteristics of male patients with *N. gonorrhoeae*-positive culture (*n* = 306) in Cambodia, January 2022–June 2023

Variable	*n* (%)
Age (years)	
<25	116 (37.9)
25–34	138 (45.1)
35–44	44 (14.4)
≥45	8 (2.6)
Mean (SD)	27.8 (7.4)
Antibiotic use (past 2 weeks)	
Yes	5 (1.6)
No	266 (86.9)
Unknown	35 (11.4)
History of travel (past 30 days)	
Yes	98 (32.0)
No	208 (68.0)
Sexual history (past 30 days)	
Sex with women only	153 (50.0)
Sex with men only	98 (32.0)
Sex with men and women	55 (18.0)
Sexual behaviours	
Vaginal sex	208 (68.0)
Anal sex	151 (49.3)
Oral Sex	23 (7.5)
Sexual partners (past 30 days)	
No more than one	113 (36.9)
Between two and five	177 (57.8)
Six or more	16 (5.2)
Median (minimum, maximum)	2 (1, 10)
STI coinfection	
HIV	55 (18.0)
Other STIs^a^	62 (20.3)
Anogenital warts	29
Syphilis	12
Genital herpes	18
Other	3

^a^Based on clinical examination and serology. Accordingly, laboratory-based diagnosis of STIs such as chlamydia, trichomoniasis or *Mycoplasma genitalium* infection remain lacking.

### AST results

The AST results for the 306 *N. gonorrhoeae* isolates are summarized in Table 3. Briefly, 15.4% of the *N. gonorrhoeae* isolates were resistant to ceftriaxone, 43.1% to cefixime, 14.4% to azithromycin and 97.1% to ciprofloxacin. Ceftriaxone-resistant *N. gonorrhoeae* isolates [*n* = 47; MIC = 0.25 mg/L (*n* = 38) or MIC = 0.5 mg/L (*n* = 9)] were collected from 8 (80%) of the 10 clinical sentinel sites, and 19 (40.4%) of these 47 ceftriaxone-resistant isolates were additionally resistant to azithromycin. High-level azithromycin-resistant isolates (MIC ≥ 256 mg/L; *n* = 40) were also obtained from 8 (80%) of the 10 clinical sentinel sites. Nineteen (6.2%) isolates were resistant to ceftriaxone, cefixime, azithromycin and ciprofloxacin and, accordingly, categorized as XDR *N. gonorrhoeae*.^24,25^ These XDR *N. gonorrhoeae* isolates (*n* = 19) were cultured in 7 (70%) of the 10 clinical sentinel sites. Furthermore, 18 (94.7%) of these XDR *N. gonorrhoeae* isolates (*n* = 19) displayed high-level azithromycin resistance (MIC ≥ 256 mg/L). However, no isolates with gentamicin MICs meeting EGASP MIC alert criteria were detected.

**Table 3.** Antimicrobial susceptibility profiles of *N. gonorrhoeae* isolates (*n* = 306) from Cambodia, January 2022–June 2023

Antimicrobials and susceptibility	*n* (%)
Ceftriaxone	
Resistant (MIC > 0.125 mg/L)^a^	47 (15.4)
Susceptible (MIC ≤ 0.125 mg/L)	259 (84.6)
Cefixime	
Resistant (MIC > 0.125 mg/L)^a^	132 (43.1)
Susceptible (MIC ≤ 0.125 mg/L)	174 (56.9)
Azithromycin	
Resistant (MIC > 1 mg/L)^b^	44 (14.4)
Susceptible (MIC ≤ 1 mg/L)	262 (85.6)
Ciprofloxacin	
Resistant (MIC > 0.06 mg/L)^a^	297 (97.1)
Susceptible (MIC ≤ 0.03 mg/L)	9 (2.9)
Gentamicin	
EGASP MIC alert value (MIC ≥ 32 mg/L)^c^	0 (0)
Susceptible (MIC < 32 mg/L)	306 (100)
Resistant to ceftriaxone, cefixime, azithromycin and ciprofloxacin (XDR NG isolates)	19 (6.2)

^a^EUCAST clinical resistance breakpoints v13.1.^23^

^b^EUCAST does not state any clinical resistance breakpoint for azithromycin, and the EUCAST ECOFF^23^ was used for azithromycin to infer resistance.

^c^EUCAST does not state any breakpoints for gentamicin and the WHO EGASP MIC alert value^19^ was instead used.

**Figure 1.**
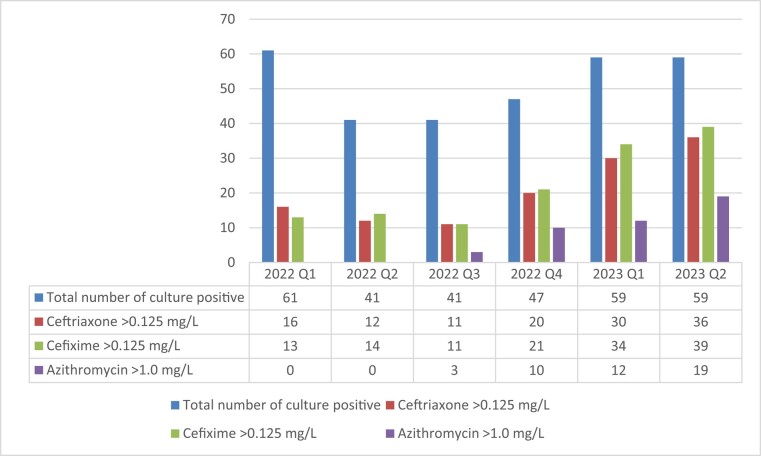
Prevalence of *N. gonorrhoeae* isolates resistant to ceftriaxone, cefixime and/or azithromycin in Cambodia, by quarter in 2022 and 2023.

Notably, from the fourth quarter of 2022, the prevalence of *N. gonorrhoeae* isolates resistant to ceftriaxone, cefixime and/or azithromycin started to substantially increase (Figure 1).

Briefly, the proportion of ceftriaxone-resistant, cefixime-resistant and azithromycin-resistant *N. gonorrhoeae* isolates increased from 26.2%, 21.3% and 0% in the first quarter of 2022 to 61.0%, 66.1% and 32.2%, respectively, in the second quarter of 2023 (Figure 1).

### Management and treatment outcomes

The most frequently reported treatment was the nationally recommended dual therapy including cefixime 400 mg plus azithromycin 1 g [200 (65.4%) cases]^21^ and the remaining cases were treated with ceftriaxone 1 g plus azithromycin 1 g [106 (34.6%) cases].

Only 30 (9.8%) of the patients returning received a TOC, and 11 (36.6%) of those patients were *N. gonorrhoeae* culture positive at TOC (urethral culture). All these 11 patients had received a first treatment with cefixime 400 mg plus azithromycin 1 g. They received a second treatment consisting of ceftriaxone 1 g plus azithromycin (1 g or 2 g) or cefixime 800 mg plus azithromycin (1 g or 2 g). The subsequent second TOC (urethral culture) was culture negative in all 11 cases.

The AST results for the 11 *N. gonorrhoeae* isolates from the 11 patients positive at TOC are summarized in Table 4.

**Table 4.** Antimicrobial susceptibility profile of *N. gonorrhoeae* isolates (*n* = 11) cultured at TOC in Cambodia, January 2022–June 2023

Isolate	MIC values (mg/L)^a^				
	Ceftriaxone	Cefixime	Azithromycin	Gentamicin	Ciprofloxacin
1	**0**.**5**	**1**	**256**	2	2
2	**0**.**125**	**0.5**	**256**	1	1
3	**0**.**125**	**0.25**	**256**	1	1
4	**0**.**25**	**1**	**256**	4	1
5	**0**.**25**	**1**	**256**	1	4
6	**0**.**125**	**0.5**	**256**	2	4
7	0.064	**0.5**	**256**	1	1
8	**0**.**125**	**1**	**256**	1	2
9	**0**.**25**	**2**	**256**	1	4
10	**0**.**125**	**1**	0.125	1	4
11	**0**.**25**	**1**	**256**	2	8
Total cases with resistance^b^	5	11	10	0	11

^a^WHO EGASP alert values are shown in bold letters.

^b^Ceftriaxone resistance: MIC > 0.125 mg/L; cefixime resistance: MIC > 0.125 mg/L; and ciprofloxacin resistance: MIC > 0.06 mg/L.^23^ Azithromycin resistance: MIC > 1 mg/L (ECOFF)^23^ used to infer resistance. Gentamicin alert value: MIC ≥ 32 mg/L.^19^

## Discussion

This is the first extensive analysis of *N. gonorrhoeae* antimicrobial susceptibility and AMR in Cambodia. From January 2022 to June 2023, high prevalences of ceftriaxone-resistant, MDR and XDR *N. gonorrhoeae* were identified in three regions, including the capital. Accordingly, the prevalence of resistance to ceftriaxone (15.4%), cefixime (43.1%) and azithromycin (14.4%) all by far exceeded the 5% AMR level used by WHO and other public health agencies to indicate when the recommended first-line empirical treatment should be changed.^3,16,17^ Consequently, it is imperative that NCHADS urgently review and update the first-line empirical gonorrhoea treatment in the National STIs guidelines in Cambodia.^21^ In Cambodia, with substantial spread of both MDR (including resistance to ceftriaxone) and XDR *N. gonorrhoeae* strains (including resistance to both ceftriaxone and azithromycin), ceftriaxone 1 g plus azithromycin 2 g dual therapy might be the only viable alternative as the recommended first-line empirical therapy for gonorrhoea. The high-dose of ceftriaxone (1 g) will be sufficient to treat many (but not all) of the ceftriaxone-resistant cases, i.e. based on the predominantly low level of ceftriaxone resistance (81% of ceftriaxone-resistant isolates had an MIC of 0.25 mg/L).^26^ Furthermore, 60% of the ceftriaxone-resistant cases were susceptible to azithromycin and will be cured by azithromycin 2 g. It is of major concern that previous national studies in Cambodia^13,14^ have identified that STI testing rates and condom use in high-risk groups are suboptimal, with recent testing and consistent condom use among female sex workers at only 40.7% and 62%, respectively. The prevalence of *N. gonorrhoeae* and vaginal discharge syndrome in this group was 18.0% and 30.4%, respectively,^13^ providing opportunities for *N. gonorrhoeae* spread, including MDR/XDR strains. Similarly, in MSM, with an *N. gonorrhoeae* prevalence of 4%, reported consistent condom use ranged between 45.6% and 51.5%.^14^ In the absence of surveys among the general population and all at-risk populations in Cambodia, the current EGASP findings of high prevalence of MDR and XDR *N. gonorrhoeae* strains, considered with the findings of previous behavioural surveillance studies,^13,14^ highlight the need to urge national stakeholders to significantly scale-up STI prevention and testing services. Crucial components of STI prevention encompass counselling and behaviour-focused strategies, which include comprehensive sexuality education, pre- and post-test counselling, guidance on safe sex practices, risk reduction counselling, and the promotion of consistent condom usage. Interventions should be directed toward high-risk groups, such as MSM, transgender individuals, sex workers and people who inject drugs. Additionally, it is essential to always inform sexual partners of current STI or HIV infection and provide them with appropriate partner management.

Worryingly, in an earlier study of female sex workers^13^ in Cambodia, regular intake of antimicrobial(s) to prevent STI was reported in 39.1% of participants. Thus, optimizing the use and regulation of antimicrobials by implementing antimicrobial stewardship at the national and healthcare facility level is critical. This includes the development of a national well-implemented action plan on *N. gonorrhoeae* AMR and AMR in general (or implementation of the global WHO action plans) with dedicated funding, development of an implementation plan along with a monitoring and evaluation frame, updating the clinical guidelines based on WHO recommendations, regulations and enforcement of prescription-only procurement of antimicrobials, and implementation of targeted awareness campaigns in schools, universities and among healthcare professionals.^27^

The limitations of the present study include that no *N. gonorrhoeae* isolates were obtained from women or from extragenital infections, such as oropharyngeal and rectal sites. Furthermore, our surveillance was conducted in 10 clinical settings across Phnom Penh (six sites) and three other provinces (Kampong Speu, Kampong Cham, and Prey Veng). Consequently, our findings may not be nationally representative of the general population in Cambodia. Currently, work is in progress to further improve and expand WHO EGASP in Cambodia. For example, it is considered to include additional clinical settings and provinces, higher proportions of populations at high risk (MSM and especially sex workers, who may not have been represented) and sampling of extragenital sites (oropharynx and rectum). Finally, the reporting coverage of demographic and behavioural information, as well as performance of TOC, requires substantial improvements.

In conclusion, a high prevalence of ceftriaxone-resistant, MDR and XDR *N. gonorrhoeae* in several cities of Cambodia were found during 2022–23 in WHO EGASP. This is of grave concern nationally in Cambodia but also from an international perspective. The wide dissemination of ceftriaxone-resistant *N. gonorrhoeae* strains [15.4% of isolates found in 8 (80%) of 10 sentinel sites] along with the high rate of cefixime resistance [43.1% of isolates found in 10 (100%) of 10 sentinel sites] stresses the urgent need to substantially scale-up and improve *N. gonorrhoeae* AMR surveillance and testing capacity, and update the first-line empirical gonorrhoea treatment in the National STIs guidelines in Cambodia.^21^ It also strongly emphasizes a need to optimize the national strategies for the general management of gonorrhoea (such as increased prevention messages and condom use), testing, treatment and follow-up of gonorrhoea cases (such as enhanced sexual contact notification and consideration of mandatory TOC), as well as improved antimicrobial prescribing policies and stewardship.^3,4,27^ From a global perspective, beside the sporadic international cases of ceftriaxone-resistant *N. gonorrhoeae* infections linked to travel to Cambodia and other Asian countries, our findings warrant the global community of STI and AMR experts, industry, scientists and scholars to urgently develop new rapid diagnostic point-of-care tests to detect *N. gonorrhoeae* (including its AMR) and new therapeutic options, e.g. the promising zoliflodacin for which the Phase 3 randomized controlled trial was finalized in 2023 (https://gardp.org/positive-results-announced-in-largest-pivotal-phase-3-trial-of-a-first-in-class-oral-antibiotic-to-treat-uncomplicated-gonorrhoea/),^28^ before global dissemination of ceftriaxone-resistant strains, some also with high-level azithromycin resistance,^5–10,17,28^ become a reality.

## Members of the WHO EGASP Cambodia working group

Phal Kun Mom (National Center for HIV/AIDS, Dermatology and Sexually Transmitted Diseases, Phnom Penh, Cambodia); Vivian Fensham (Freelance laboratory support, consultant of the WHO); Ellen Kersh (Division of STD Prevention, Centers for Disease Control and Prevention, Atlanta, GA, USA); Philippe Cavailler and Yamuna Mundade (Global HIV, Hepatitis and STI Programmes, WHO, Geneva Switzerland); Sebastiaan J. van Hal, Ratan L Kundu and Tiffany R. Hogan (WHO Collaborating Centre for Sexually Transmitted Infections and Antimicrobial Resistance, New South Wales Health Pathology, Microbiology, The Prince of Wales Hospital Randwick, NSW, Australia); David M. Whiley (Faculty of Medicine, University of Queensland, Brisbane, Australia); and Kiyohiko Izumi and Takeshi Nishijima (WHO, the Western Pacific Regional Office).
